# Typhoid Fever

**Published:** 1913-09

**Authors:** 


### BUFFALO MEDICAL JOURNAL

**A Monthly Review of Medicine and Surgery**

EDITOR AND PUBLISHER DR. A. L. BENEDICT, 228 Summer St., corner of Elmwood Ave., Buffalo. (Address for all communications. Please make personal and telephone calls before 1 P. M.)

THE BUFFALO MEDICAL JOURNAL is the third, in age, in the western continent. It is independent but supports professional organization. As a scientific medium it is unrestricted in territory. As a news medium. it is limited mainly to the western four districts of the Medical Society of the State of New York. The co-operation of physicians is asked in securing personal, obituary and other news items. Secretaries of Medical Societies in this region are requested to send brief reports of meetings. Full transactions will be published at cost of composition.

Subscription $2.00 a year in advance; $3.00 for two years in advance. Changes and errors in address or failure or duplication of delivery, should be reported immediately. Back numbers will be supplied. if possible but requests for copies should be made promptly,

YEARLY VOLUME 69 SEPTEMBER, 1913 No. 2

### Typhoid Fever

This is the season at which typhoid fever is particularly likely to be encountered. For some years we have held the opinion, which is becoming less heterodox, that the seasonal prevalence of typhoid is not due to strictly natural phenomena but to the relative increase of exposure to miscellaneous, unguarded and sometimes obviously contaminated water supplies, during the vacation season. While the possibility of conveyance by ice, milk, fresh vegetables, flies and other insects and by the soiled clothing and hands of "carriers," or by analogous routes from acute cases, latent or recognized, deserves practical consideration, evidence supports the older belief that the principal source of typhoid is drinking water into which typhoid bacilli have entered, usually from faeces and urine—the latter being infected in about 20 per cent. of samples examined.

While the possibility of typhoid-like fevers from virulent and comparatively massive infection with colon bacilli and various strains of bacilli intermediate between the colon and the true typhoid bacillus must be considered, and while no one will advocate the discharge of sewage into potable water, it is well to place clearly before the medical profession, if not the laity, the fact that typhoid arises only from typhoid and not from sewage contamination in and of itself. We understand that, except in experiments with intentionally infected water, the typhoid organism has been identified in water, only once or twice. Warnings as to the state of the water supply are occasionally published in which it is said that typhoid bacilli are present. Accuracy in such warnings is important, not only because, in general, it is a good rule to tell the truth, but because, such warnings, repeated, and without the consequences predicted, tend to establish a state of skepticism and carelessness on the part of the public. Inaccurate statements emanating from one place obviously tend to produce inaccurate wording of newspaper warnings in others and thus to discredit conservative statements by other boards of health.

Excepting in an experimental sense, typhoid is almost if not quite the nearest approach to a purely human disease. Hence, the danger of infection is enormously multiplied by the occurrence of even a small discharge of contaminated material from a single human case into a water supply and, conversely, is greatly diminished—and theoretically by geometric and not arithmetic progression—by even a moderate diminution of existing cases. The relative extent of an epidemic of typhoid is inversely proportionate to the bulk of the aggregate water supply and, hence, usually, to the size of the community itself, although a small community drawing from a large river or lake would show only the same proportionate incidence as a large one. It is, of course, impossible to give anything approaching accurate percentages of incidence with relation to population, but the following illustrations have been supported by experience many times: When the community consists of a single family and the water supply of a well, the leakage into the well of even a small amount of the discharges of a single patient, may produce an epidemic of 100 per cent.; when the community consists of a few hundred or thousand persons and the water supply consists of springs, a small lake or creek, one patient may set up an epidemic of 10 per cent. to 50 per cent.; in large cities, drawing water from a river or lake capable of supplying many times the population, even if the contamination is notoriously high, we seldom encounter for a whole year a typhoid mortality of more than 200 per 100,000 population, which corresponds to an incidence of 2 per 1000 on the basis of a 10 per cent. average mortality or of 4 per cent. on the basis of a failure to report half the cases. It should be remembered that even 4 per cent. of incidence for a large city is high, and that it covers an entire year, whereas the preceding figures apply to single epidemics covering a few weeks.

To take a purely local example, we believe that the improvement in the water supply of Niagara Falls is already having a marked influence on the general incidence of typhoid. This city had, for many years a high typhoid incidence, although not excessively so as compared with other cities. But, somewhere near a twelfth of its population was being immunized against further typhoid infection yearly and the more intelligent part of the population was being warned by precept and example, constantly, against using the municipal water supply in a raw state. Hence, it is fair to assume that the infectivity of the water supply was considerably greater than was indicated by the typhoid mortality. About a million persons visit Niagara Falls annually, mainly in the summer, when thirst overcomes caution. While it is impossible to present any statistics, it is obvious that many of these visitors must have become infected and must have originated new centers of infection in their own homes. The same process of education and sanitary inforcement that has diminished the danger of the Niagara Falls water has been operative at thousands of points more or less sought by tourists or travelers on business. A quarter of a century ago there was scarcely a large city or place of interest in Europe and comparatively few in America which did not have a high typhoid incidence, according to modern standards. Today, one can drink the water of almost every such place in western Europe with a feeling of security and one can count on his fingers the cities of a hundred thousand population or more in the United States in which the typhoid death rate exceeds 50 per 100,000. In most of such cities the typhoid death rate is at or below 20, and probably at least half of this is due to other sources than the drinking water of the city itself.

Based on purely bacteriologic evidence of the survival of typhoid bacilli in natural environments, and, for the moment ignoring carriers, one might almost say that if all cases of human typhoid could be done away with or absolutely isolated for a year, the utmost carelessness with regard to sewerage and water supplies could be indulged in, with impunity. We hasten to disclaim the advocacy of such a course but, from the academic standpoint, the proposition is interesting. Moreover, there is good archaeologic evidence that such a condition existed in America prior to its settlement by Europeans and there is no historic evidence that typhoid existed until it was introduced from Europe.

We do wish, however, to emphasize the optimistic contention that, just as soon as the incidence of typhoid—or any other infection— has been diminished past the point at which a superabundance of possible sources of infection exists, every case prevented, by any means and anywhere, tends, on the average to produce a further reduction of incidence not of one but of several cases and that this diminishing effect proceeds by geometric progression. There is considerable evidence that we have already reached the point at which carelessness in sanitary matters fails to produce its logical results, because carefulness at some other point has prevented a potential source of infection.

On the other hand, the less typhoid occurs, the greater danger is there of a massive epidemic if a single focus of the disease develops, on account of the lack of immunization by pre-existing high incidence. This fact was well exemplified in the Spanish-American War. Indeed, in many regiments it appeared that almost every man who had not previously had the fever, contracted it in camp. But, fortunately, recent experience with artificial immunization has shown that this result can be avoided, to a large degree. This immunization, important from the military standpoint and from that of the individual, is even more important from that of the whole population for it prevents the development of foci in which typhoid bacilli are multiplied in numbers and increased in virulence.

Most of the cities and towns of our territory have fairly good water supplies. In many instances we believe that these water supplies are even safer than appears from typhoid statistics. It is of the utmost importance to know not only how many cases of typhoid occur in a given city or town, but what proportion of these cases occur in residents and what proportion are due to local factors and what to imported infection. Statistics of this nature carefully collated by practitioners from study of cases and intelligently grouped by central authorities have a value that can be expressed in dollars and cents. For example, there is no need for a city to spend a million dollars to improve its water supply simply because, as a matter of humanity, it is treating grave cases of typhoid brought in from other places or because its own citizens drink from contaminated wells at picnics or come back from vacations with typhoid incurred by the sanitary deficiencies of summer resorts.

Some years ago our Associate Editor, Prof. Pel, gave a most significant reply to a question as to why malaria was so uncommon in Holland, in spite of the marshy nature of so much of the ground. He said that the anopheles was common but that malaria had been practically exterminated by quinine administered to patients. At first thought, this application of the "one man at a time" doctrine, seemed far fetched. But a little thought will convince the most skeptic that it is good doctrine in medicine as well as in religion. The same principle applies to typhoid. Each physician, in attendance on even a suspected case of typhoid, must realize his grave responsibility not only to the patient but to the community. He is guilty of complicity in manslaughter if he does not rigorously carry out details of prophylaxis which shall render that particular case inocuous to the community. One may go farther and say that the local health department is at fault if it does not, in some way, provide means for determining with scientific accuracy, when the period of quarantine may be concluded.

This brings us to the consideration of typhoid carriers, too difficult a subject to be discussed here.

